# Local Tramadol Improves the Anesthetic Success in Patients with Symptomatic Irreversible Pulpitis: A Meta-Analysis

**DOI:** 10.3390/healthcare10101867

**Published:** 2022-09-25

**Authors:** Eduardo Gómez-Sánchez, Lorenzo Franco-de la Torre, Ronell Eduardo Bologna-Molina, Nelly Molina-Frechero, Nicolás Addiel Serafín-Higuera, Adriana Hernández-Gómez, Ángel Josabad Alonso-Castro, Daniel Sat-Muñoz, Mario Alberto Isiordia-Espinoza

**Affiliations:** 1 Departamento de Ciencias Fisiológicas, División de Disciplinas Básicas para la Salud, Cuerpo Académico Ciencias Morfológicas en el Diagnóstico y Tratamiento de la Enfermedad (UDG-CA-874), Centro Universitario de Ciencias de la Salud, Universidad de Guadalajara, Guadalajara 44340, Jalisco, Mexico; 2 Instituto de Investigación en Ciencias Médicas, Departamento de Clínicas, División de Ciencias Biomédicas, Cuerpo Académico Terapéutica y Biología Molecular (UDG-CA-973), Centro Universitario de los Altos, Universidad de Guadalajara, Tepatitlán de Morelos, Guadalajara 47620, Jalisco, Mexico; 3 Área de Patología Bucal, Facultad de Odontología, Universidad de la República (UDELAR), Montevideo 11600, Uruguay; 4 Departamento de Salud, Laboratorio de Cariología y Medicina Oral, Universidad Autónoma Metropolitana-Xochimilco, Mexico City 04960, Mexico; 5 Facultad de Odontología, Universidad Autónoma de Baja California, Mexicali 21040, Baja California, Mexico; 6 Departamento de Ciencias de la Salud, División de Ciencias Biomédicas, Centro Universitario de los Altos, Universidad de Guadalajara, Tepatitlán de Morelos 47620, Jalisco, Mexico; 7 Departamento de Farmacia, División de Ciencias Naturales y Exactas, Universidad de Guanajuato, Guanajuato City 36050, Guanajuato, Mexico

**Keywords:** tramadol, symptomatic irreversible pulpitis, anesthesia success rate, pain control, adverse effects

## Abstract

Symptomatic irreversible pulpitis is a painful clinical condition with a broad inflammatory component. Dental anesthesia in these patients is affected by the inflammatory process, reporting a high incidence of anesthesia failure. The aim of this systematic review and meta-analytical evaluation was to determine the effect of pre-treatment with tramadol in patients with symptomatic irreversible pulpitis, as well as for pain control and adverse effects. This study was registered in PROSPERO (ID: CRD42021279262). PubMed was consulted to identify clinical investigations comparing tramadol and placebo/local anesthetics in patients with symptomatic irreversible pulpitis. Data about the anesthesia, pain control, and adverse effects were extracted. Both the anesthetic success index and the adverse effects of local tramadol and placebo were compared with the Mantel–Haenszel test and odds ratio. Data analysis showed that the local administration of tramadol increased the anesthetic success rate when compared to placebo in patients with symptomatic irreversible pulpitis (*n* = 228; I^2^ = 0; OR = 2.2; 95% CIs: 1.30 to 3.79; *p* < 0.004). However, local administration of tramadol increased the risk of adverse effects when compared to placebo/local anesthetics (*n* = 288; I^2^ = 0; OR = 7.72; 95% CIs: 1.37 to 43.46; *p* < 0.02). In conclusion, this study shows that the local administration of tramadol increases the anesthetic success index when compared to placebo in patients with symptomatic irreversible pulpitis.

## 1. Introduction

Patients with symptomatic irreversible pulpitis (SIP) face an inflammatory condition with moderate to severe pain that must be treated as a dental emergency. In addition, these patients have pain during root canal treatment due to the anesthetic agents’ present difficulty to carry out dental pulp anesthesia [1,2,3]. 

Non-steroidal anti-inflammatory drugs (NSAIDs) [4] and glucocorticosteroids [5], as well as, to a lesser extent, opioid analgesics have been used to improve the anesthetic success in patients with SIP [6,7,8]. These kinds of drugs suppress the inflammatory process—NSAIDs and glucocorticosteroids—[4,5] or, in the case of opioid analgesics, hyperpolarize the membranes of the nerve fibers—a traditional opioid mechanism of action—which would translate into a decrease in the perception of pain [9,10,11,12]. In both cases, the goal of the preoperative administration of a drug would be to obtain deeper anesthesia to treat patients with this clinical condition [4,5,9,10,11,12].

In this regard, the opioid analgesic most often used in patients with SIP has been tramadol, which has been used in some clinical trials for improving anesthesia success and for postoperative pain control [13,14,15,16,17,18]. However, the efficacy of tramadol in the dental field has been questioned because no benefits were found when compared to NSAIDs after third molar surgery. Furthermore, tramadol increases the number of adverse effects in comparison to NSAIDs [19].

Tramadol binds to µ-opioid receptors and inhibits monoamine reuptake to exert its therapeutic effect and side effects. Compared to other opioids, it has a low potential for abuse and a low incidence of adverse effects [20]. It has rapid absorption and distribution with maximum serum concentration reached after 2 h [21]. It is metabolized in the liver and its main route of excretion is the kidneys. A small amount of tramadol crosses the placental barrier, and, similarly, a small portion of the drug is excreted in breast milk [21,22]. Several local mechanisms of action of tramadol have been demonstrated, which could be the key to understanding the effect in patients with SIP [17]. An anesthetic effect due to sodium channel blockade [23,24], an anti-nociceptive effect due to potassium channel blockade [23,25] and analgesia acting on peripheral opioid receptors [23,26,27,28] are the local mechanisms that could be involved in the increased efficacy of anesthetic blockade in patients with SIP. 

For this reason, we conducted this systematic review and meta-analytic evaluation (SRME) to determine anesthetic success when using tramadol as a pretreatment in patients with SIP, as well as pain control and adverse effects.

## 2. Materials and Methods

### Study Design

This SRME was completed at the Instituto de Investigación en Ciencias Médicas from the Centro Universitario de los Altos of the Universidad de Guadalajara following PRISMA guidelines [29,30] and it has a record in the National Institute for Health Research from the University of York (PROSPERO ID: CRD42021279262).

## 3. Information Search

The keywords used to perform the PubMed searches and identification of published articles were: “tramadol”, “symptomatic irreversible pulpitis”, “active dental pain”, “dental pain”, “endodontic treatment”, and “root channel therapy”. In the same way, three screens were employed: 1. Article kind: clinical trial or randomized controlled trial; 2. Language: English and Spanish; and 3. Species: humans. The article search was carried out from 15 January 2022 to 15 March 2022. 

### 3.1. Population, Interventions, Control, and Outcome (PICO) Approach [31]

#### 3.1.1. Inclusion Criteria

Population: randomized, double-blind, clinical trials.

Interventions: tramadol administration in patients with SIP.

Control: a placebo group or a local anesthetic group.

Outcomes: anesthetic success, anesthesia depth, anesthetic time, pain intensity, rescue analgesic intake, and adverse effects.

#### 3.1.2. Exclusion Criteria

Clinical studies reporting a loss to follow-up of more than 20%.

High risk of bias according to the Cochrane Collaboration’s risk-of-bias tool.

### 3.2. The Cochrane Collaboration’s Risk-of-Bias Tool

The evaluation was completed across the seven points of the original tool: (1) random sequence generation; (2) allocation concealment; (3) masking (blinding of participants and personnel); (4) blinding outcome assessment; (5) incomplete outcome data; (6) reporting bias; and (7) other bias [32,33,34]. Each point was qualified into three categories: low risk, medium risk, and high risk (green, yellow, or red color, respectively) [32,33,34].

### 3.3. Extraction of Information

Article ID data, experimental design, treatment groups, size sample (*n*), dose and route of administration, anesthetic success index, anesthesia depth, anesthetic time, pain intensity, rescue analgesic intake, and adverse effects were obtained.

Aksoy and Ege published two articles using local tramadol in patients with SIP in 2020. These reports were placed with a lowercase letter to identify them in this SRME (“Aksoy and Ege, 2020a” [13] and “Aksoy and Ege, 2020b” [14]).

Two independent clinical researchers made the bias measurement and data extraction. The differences between them were decided by a third investigator, only when necessary.

### 3.4. Statistical Analysis

The review management 5.3 software for Windows from the Cochrane Library (London, UK) was used for data analysis. Both anesthetic success index and adverse effects of local tramadol versus placebo/local anesthetic were compared with the Mantel–Haenszel test and odds ratio (OR). Data inconsistency was analyzed with the I^2^ test. Furthermore, the funnel plots were employed to assess the publication bias of the included clinical studies. A *p*-value ≤ of 5% (0.05), with an OR ≥ 1 within a 95% confidence interval (CIs), was considered a statistical difference [32,35,36,37,38].

## 4. Results

### 4.1. Search and Measurement of Bias

**Figure 1:**
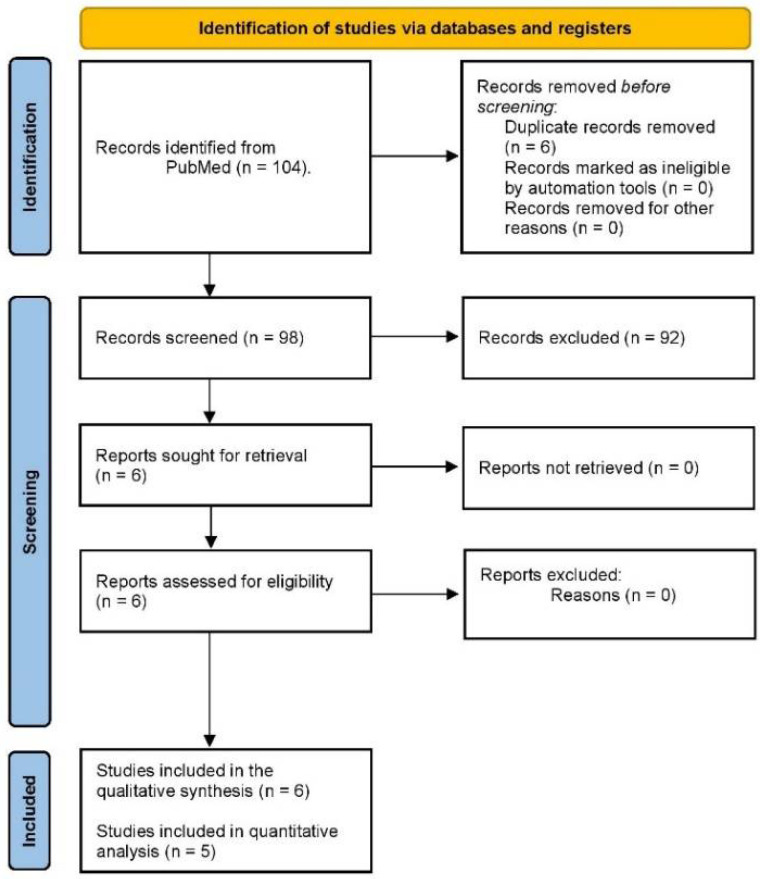
Study flow chart.

**Figure 2:**
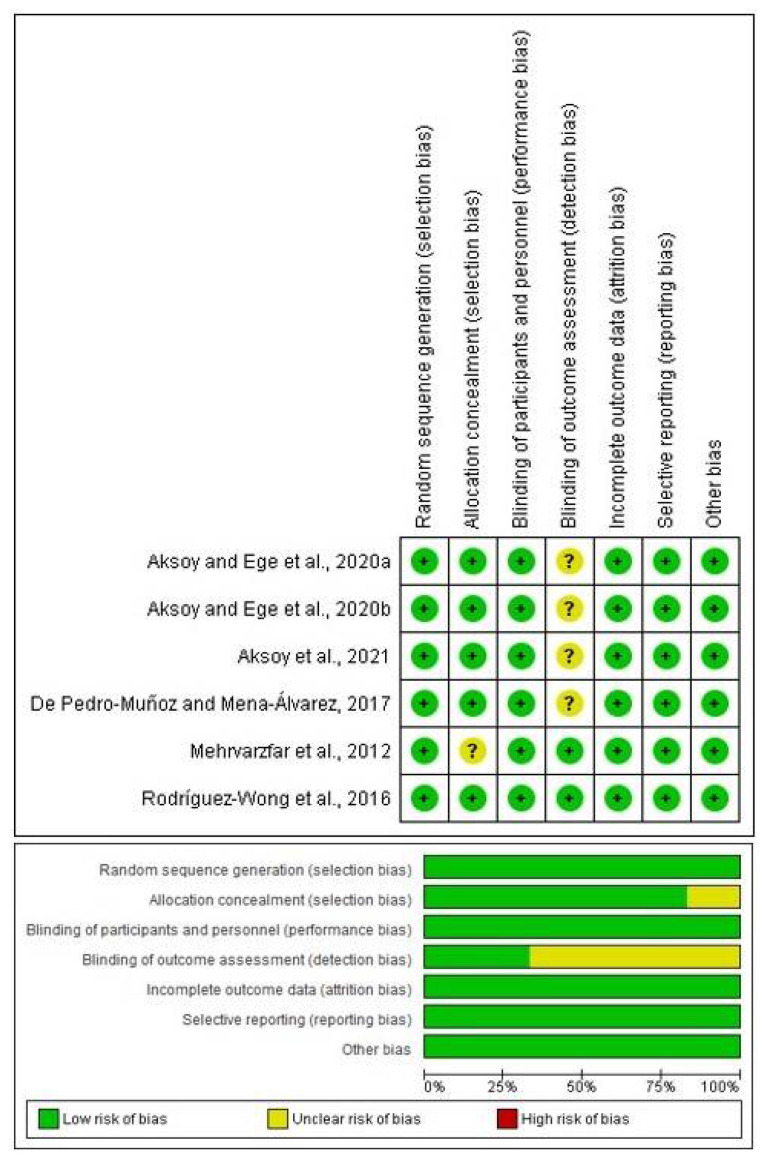
Risk of bias assessment [13,14,15,16,17,18].

The initial PubMed search showed a total of 104 articles using tramadol in dentistry, of which six articles compared tramadol and placebo/local anesthetics in patients with SIP (Figure 1). Moreover, all clinical investigations had a low risk of bias according to the bias tool used (Figure 2).

### 4.2. Qualitative Assessment

Five out of six articles included molars and one-sixth included anterior teeth with SIP. The most used anesthetic agent in the included articles was 4% articaine and 1:100,000 (1/6) [16] or 1:200,000 adrenaline (2/6) [14,15]; this was followed by 2% lidocaine—1:80,000 adrenaline (2/6) [13,18], and 2% mepivacaine—1:100,000 adrenaline (1/6) [17]. On the other hand, 2/2 studies showed that the local administration of tramadol was more effective for pain control than placebo [14,18]. Furthermore, 1/4 clinical investigations reported that local tramadol increased the anesthetic success index in comparison to placebo [17]. Details of the included studies are presented in Table 1.

### 4.3. Quantitative Evaluation

**Figure 3:**
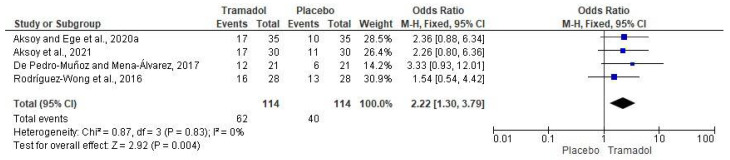
Forest plot of the effect of local tramadol versus placebo/local anesthetics on anesthetic success [13,15,16,17].

The evaluation of the anesthesia success rate of submucosal tramadol versus placebo was performed with four clinical investigations (*n* = 228) [13,15,16,17]. Data analysis showed that the local administration of tramadol increased the anesthetic success rate when compared to placebo/local anesthetics in patients with SIP (*n* = 228; I^2^ = 0; OR = 2.2; 95% CIs: 1.30 to 3.79; *p* < 0.004; Figure 3).

**Figure 4:**
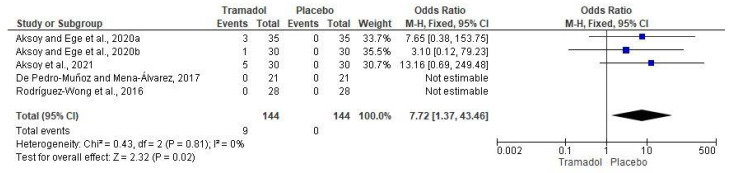
Pooled analysis on adverse effects of local tramadol and placebo/local anesthetics [13,15,16,17].

The adverse effects assessment of local tramadol and placebo was made using data from five clinical trials (*n* = 288) [13,15,16,17]. In this regard, nine patients who were given local tramadol presented adverse effects, while in the placebo/local anesthetics group no patients reported adverse effects (*n* = 288; I^2^ = 0; OR = 7.72; 95% CIs: 1.37 to 43.46; *p* < 0.02; Figure 4).

### 4.4. The Publication Bias

**Figure 5:**
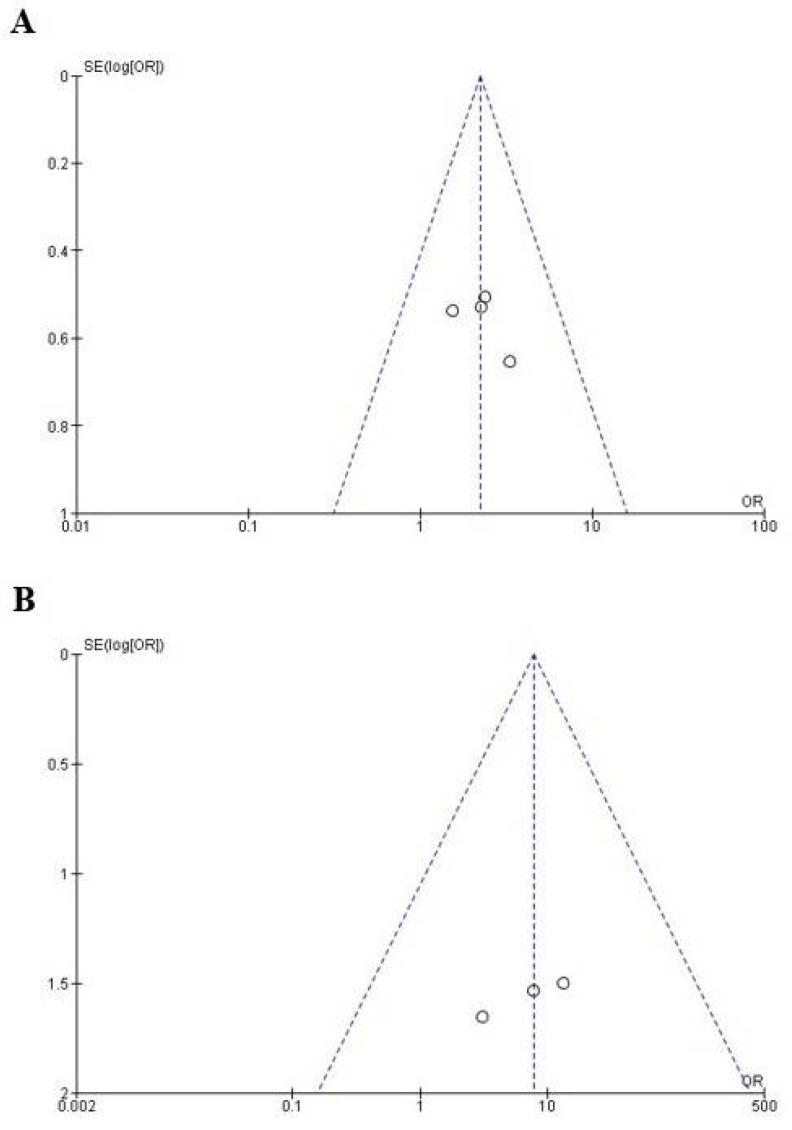
Evaluation of publication bias of the anesthetic success (**A**) and adverse effects (**B**) variables.

The publication bias of the published clinical investigations was evaluated using the data of anesthetic success (Figure 5A) [13,15,16,17] and pain control (Figure 5B) [13,14,15,16,17] of tramadol and placebo/local anesthetic in patients with SIP. In both cases, the data fell within the funnel plot, indicating a low risk of publication bias.

## 5. Discussion

The most important clinical result of this SRME was that the local administration of tramadol increases the anesthetic success index when compared to placebo/local anesthetic in patients with SIP. Moreover, according to the findings of this SRME, local tramadol increases the risk of adverse effects in comparison to placebo. We consider that the risk/benefit balance justifies the use of local tramadol as the high anxiety, severe pain, and suffering that patients with this clinical condition [1,2,3]. We could consider the use of local tramadol as an experimental procedure rarely used in daily practice. Generating more information on the efficacy or otherwise of tramadol in patients with SIP could help introduce or rule out this pharmacological procedure.

The bias tool showed that only one global and individual indicator, detection bias, was rated at medium risk of bias (blinding of outcome assessment), while the rest of the indicators showed a low risk of bias (Figure 2). In addition, the publication bias of the included studies using tramadol to improve dental anesthesia in patients with SIP was low because all points (representing each individual study) on the funnel plot are within the limits that make up the graph. Only 1/4 of the published studies using tramadol to improve dental anesthesia showed positive results and three clinical trials found no statistical difference between tramadol and placebo/local anesthetic. In other words, most of the published reports present negative results [39,40,41].

The local administration of tramadol in dentistry has been shown to improve the anesthetic and analgesic effects of different agents [42]. In this regard, tramadol has been used as a local anesthetic for dental extraction of upper teeth and it is recommended when patients cannot receive a conventional anesthetic agent [43]. Moreover, the local administration of tramadol increased the anesthetic activity of 4% articaine and 1:100,000 adrenaline following third molar removal [44,45]. However, these findings are different from those reported by Ceccheti et al., 2014, who found that submucosal tramadol improved analgesia but did not extend the anesthetic activity of mepivacaine 2%—1:20,000 levonorfedrine after third molar surgery [46]. Additionally, it has been reported that submucosal tramadol increased anesthesia depth by 2% mepivacaine with 1:100,000 adrenaline in healthy teeth [47]. Moreover, the submucosal administration of tramadol and oral ketorolac produced a better analgesic effect than oral ketorolac plus submucosal placebo after third molar surgery [48]. In addition, the local application of tramadol was effective for pain control after third molar surgery [49]. In this SRME, only one clinical trial reported an increase in the anesthetic time of mepivacaine by using local tramadol in patients with SIP [17].

One point that strongly draws attention to tramadol is the action that this drug induced on a peripheral nerve, a local anesthetic-like effect. Tsai et al., 2001, reported that the local administration of tramadol on the sciatic nerve blocks the spinal somatosensory potentials in rodents [50]. Altunkaya and collaborators evaluated the anesthetic activity of tramadol in different clinical procedures—cutaneous lesions surgery and tendon repair surgery—and concluded that tramadol has an anesthetic action similar to prilocaine 2% [51] or lidocaine with epinephrine [52]. Moreover, tramadol presents additional mechanisms, as explained in the introduction [23,24,25,26,27,28]. All these mechanisms of anesthetic and anti-nociceptive action of tramadol could be acting on specific receptors in the dental pulp to improve the local anesthetic effect in patients with SIP.

The adverse effects of tramadol have been evaluated in other systematic reviews and meta-analyses. Tsaousi et al., 2020 found a negligible association of adverse effects after local administration of tramadol in children undergoing tonsillectomy [53]. In this sense, Mattar et al., 2019, carried out the evaluation of analgesia and adverse effects of tramadol during a diagnostic outpatient’s hysteroscopy and reported that the pooled analysis showed no increase in the risk of minor adverse effects, such as nausea, vomiting, and bradycardia [54]. Moreover, Isiordia-Espinoza et al., 2014, informed that tramadol increased the risk of adverse effects on the nervous system as nausea and dizziness when compared to NSAIDs after third molar surgery [19]. In this SRME, 9/144 patients in the tramadol group had a significantly increased risk of adverse effects when compared to the control group. Nausea and dizziness were the adverse effects reported by patients receiving tramadol in the clinical trials included in this SRME. However, the upper ICs is wide, which could indicate that the significant data did not have great relevance, despite the OR obtained.

The adherence to international guidelines for conducting this SRME, the use of studies with a low risk of bias, and the low heterogeneity of the data are the main strengths of this study. However, some weaknesses were detected, for example, the low number of studies detected, a relatively small sample size, the different doses of tramadol used, the different methods employed to assess the same variable (i.e., the pain intensity was evaluated with the 100 mm VAS in some clinical trials and in other studies with the 170 mm Heft-Parker VAS), and the lack of reported data as the standard deviation, which avoids a study being included in the pooled analysis.

The most important finding of this SRME was that when pooled analysis of these data was performed, the anesthetic success rate showed a statistical difference in favor of local administration of tramadol versus placebo/local anesthetics in patients with SIP, highlighting two important aspects: the increase in the sample size and the power of the statistical tests (Figure 3).

In conclusion, this SRME demonstrates that the administration of submucosal tramadol increases the anesthetic success rate with minor adverse effects in comparison to placebo/local anesthetics in patients with SIP undergoing root channel treatment.

## Figures and Tables

**Table 1:** Identification of study, study design, treatments (*n*), patients, dental procedure, clinical evaluation, and conclusion of the included studies.

ID Study and, Study Design	Treatments (*n*)	Details of Patients, Dental Procedure, and Evaluation	Conclusions
Aksoy and Ege, 2020a [13]. Randomized, double-blind, parallel, clinical study.	Group A: Tramadol 100 mg (*n* = 35). Group B: Lidocaine 40 mg (*n* = 35). Group C: Normal saline (*n* = 35). All treatments were given (2 mL volume) across the mucobuccal fold of the mandibular molar.	Patients aged 18 to 60 years with symptomatic irreversible pulpitis diagnosis (moderate to severe pain) and, without periapical radiolucency on radiography at a mandibular molar were included. Patients without analgesic medication, at least, 24 h before the study. Positive Endo Ice F cold tests. A standard cartridge with 1.8 mL 2% lidocaine with 1:80,000 epinephrine was administered via the IANB route. Anesthesia was successful when the pain level of patients was no pain or mild pain. The anesthesia success rate and adverse effects were evaluated.	The results did not show any statistical difference between groups.
Aksoy and Ege, 2020b [14]. Randomized, double-blind, parallel, clinical trial.	Group A: Tramadol 100 mg (*n* = 30). Group B: Dexamethasone 8 mg (*n* = 30). Group C: Normal saline (*n* = 30). All treatments were given (2 mL volume) across the mucobuccal fold of the mandibular molar.	Healthy patients aged 18 to 65 years old with a diagnosis of symptomatic irreversible pulpitis (moderate to severe pain) in a mandibular molar, radiographically normal periapical area, and no pain on percussion were included. Patients without analgesic medication, at least, 12 h before the study. Positive Endo Ice F cold tests. An IANB using 4% articaine with 1:200,000 epinephrine was used. Postoperative pain intensity, rescue analgesic medication, and adverse effects were evaluated.	Submucosal tramadol was most effective for pain control when compared to saline.
Aksoy et al., 2021 [15]. Randomized, double-blind, parallel, clinical assay.	Group A: Tramadol 100 mg (*n* = 30). Group B: Dexamethasone 8 mg (*n* = 30). Group C: Articaine 4% (*n* = 30). Group D: Normal saline (*n* = 30). All treatments were given (2 mL volume) across the mucobuccal fold of the mandibular molar.	Healthy patients aged 18 to 65 years old with a diagnosis of symptomatic irreversible pulpitis (moderate to severe pain) in a mandibular molar, radiographically normal periapical area, and no pain on percussion were included. Patients without analgesic medication, at least, 24 h before the study. Positive Endo Ice F cold tests. An IANB using 4% articaine with 1:200,000 epinephrine was used. Anesthesia was successful when the pain level of patients was no pain or mild pain. Sensory blockade, duration of anesthesia, anesthetic success index, and adverse effects were assessed.	Submucosal articaine increased the success anesthesia rate and dexamethasone the duration of the anesthetic activity when compared to saline in patients with symptomatic irreversible pulpitis.
De Pedro-Muñoz and Mena-Álvarez, 2017 [16]. Randomized, double-blind, parallel, clinical investigation.	Group A: Tramadol 50 mg (*n* = 21). Group B: Normal saline (*n* = 21). All treatments were given (1 mL volume) across the mucobuccal fold of the mandibular molar.	Patients aged 18 to 64 years with symptomatic irreversible pulpitis diagnosis in a mandibular molar. Patients without analgesic medication, at least, 24 h before the study. Positive Endo Ice F cold tests. A standard cartridge with 1.8 mL 4% articaine with 1:100,000 epinephrine was administered via the IANB route. The access cavity, the anesthesia success rate, and adverse effects were evaluated.	Submucosal administration of tramadol increased the success rate of access cavity in patients with symptomatic irreversible pulpitis.
Mehrvarzfar et al., 2017 [18]. Randomized, double-blind, parallel, clinical study.	Group A: Tramadol 100 mg (*n* = 24). Group B: Acetaminophen 375 mg (*n* = 23). Group C: Naproxen 500 mg (*n* = 24). Group D: Placebo (*n* = 24). All treatments were administered orally.	Patients aged 20 and 60 years old without systemic illness and, no pregnant women. Patients without analgesic medication, at least, 12 h before the study. Anesthesia was done using 1 cartridge of lidocaine and adrenaline 1:80,000. Pain intensity was assessed pre-operatively, and at 6, 12, and 24 h. Adverse effects were not evaluated.	Tramadol was more effective for pain control after root channel therapy.
Rodríguez-Wong et al., 2016 [17]. Randomized, double-blind, parallel, clinical trial.	Group A: A cartridge with 1.3 mL of 2% mepivacaine with epinephrine 1:100 000 plus 0.5 mL of tramadol 25 mg/mL (*n* = 28). Group B: A cartridge with 1.8 mL of 2% mepivacaine with epinephrine 1:100 000 (*n* = 28). All treatments were given (1.8 mL volume) across the mucobuccal fold of the mandibular molar.	Patients aged 18 years or older with symptomatic irreversible pulpitis in a mandibular molar. Patients without analgesic medication, at least, 12 h before the study. Positive Endo Ice F cold tests. The IANB was performed according to the information of treatment groups. Anesthesia was successful when the pain level of patients was no pain or mild pain. Sensory blockade, duration of anesthesia, anesthetic success index, and adverse effects were assessed.	There was no statistical difference between treatment groups.

## Data Availability

Data are contained within the article.
